# Frailty is a predictor of moderate to severe pain after robot‐assisted laparoscopic prostatectomy: A case‐control study (FRAP study)

**DOI:** 10.1002/bco2.17

**Published:** 2020-05-14

**Authors:** Masaki Momota, Shingo Hatakeyama, Osamu Soma, Itsuto Hamano, Naoki Fujita, Teppei Okamoto, Kyo Togashi, Tomoko Hamaya, Tohru Yoneyama, Hayato Yamamoto, Takahiro Yoneyama, Yasuhiro Hashimoto, Chikara Ohyama

**Affiliations:** ^1^ Department of Urology Hirosaki University Graduate School of Medicine Hirosaki Japan; ^2^ Department of Advanced Blood Purification Therapy Hirosaki University Graduate School of Medicine Hirosaki Japan; ^3^ Department of Advanced Transplant and Regenerative Medicine Hirosaki University Graduate School of Medicine Hirosaki Japan

**Keywords:** frailty, geriatric 8, prostate cancer, pain, prostatectomy

## Abstract

**Objective:**

To investigate the association of pain with frailty in patients with localized prostate cancer (PC) who underwent robot‐assisted laparoscopic radical prostatectomy (RARP).

**Materials and Methods:**

Between January 2017 and June 2019, we prospectively evaluated the geriatric 8 (G8) score, simplified frailty index (sFI), and numerical rating scale (NRS) of 154 patients with localized PC who underwent RARP at our institution. NRS was measured on preoperative day 0, postoperative days 1, 2, 3, and at discharge. Moderate to severe pain was defined as NRS ≥ 5, whereas frailty was defined as G8 ≤ 14. The primary objectives of this study were to investigate the effects of moderate to severe pain (NRS ≥ 5) on frailty, postoperative complications, and the use of analgesics after RARP. Our secondary objectives were the effect of frailty on postoperative complications and the use of analgesics.

**Results:**

The median age of participants was 69 years. Of 154 patients, 37 (24%) and 61 (40%) were classified to have NRS ≥ 5 and G8 ≤ 14, respectively. Patients with NRS > 5 presented significantly association with G8 < 14, whereas they did not show the association with sFI, complication, or analgesics. Multivariate logistic regression analysis showed that G8 ≤ 14 was significantly associated with NRS ≥ 5. Frailty was not significantly associated with postoperative complications and analgesics.

**Conclusions:**

Frailty was significantly associated with moderate to severe pain after RARP, and might be a potential predictor of postoperative pain. Frail patients require individual care to avoid painful experiences.

## 1 INTRODUCTION

Reflecting an increased focus on elderly patients with urological cancers, interest in frailty has also gained increasing prominence.^1^, ^2^, ^3^, ^4^, ^5^, ^6^ Although frailty screening in patients with cancer has clinical value, there is not enough evidence available for the optimal screening tool and clinical implication of frailty on postoperative outcomes in prostate cancer (PC).^7^, ^8^, ^9^ PC is the most frequently diagnosed male cancer in the United States, Europe, and Japan,^10^, ^11^, ^12^, ^13^, ^14^, ^15^ and the treatment requires concerns of frailty because health status including frailty should influence the treatment outcomes.^16^, ^17^ Several simple assessment tools are available, including simplified frailty index (sFI)^18^ and the geriatric 8 (G8) screening tool.^19^ A recent systematic review found that although G8 was one of the most robust screening tools currently available, it was also associated with treatment‐related complications.^20^ As frailty was suggested to be associated with unfavorable outcomes in urologic surgeries,^7^, ^8^, ^9^, ^18^ we hypothesized that patients with frailty might experience greater pain compared with patients without frailty. However, no evidence is available for the effect of frailty on postoperative pain in patients with localized PC who have undergone robot‐assisted laparoscopic radical prostatectomy (RARP). Therefore, we performed a prospective observational study investigating the effect of G8 on postoperative pain in this particular group of patients.

## 2 MATERIALS AND METHODS

### 2.1 Ethics statement

This study was performed in accordance with the ethical standards established by the Declaration of Helsinki and was approved by the ethics review board (authorization number: 2014‐297). All participants provided written or verbal informed consent. The study was registered on the UMIN‐CTR (UMIN000038969).

### 2.2 Study population and treatment protocol

This prospective observational study planned to enroll 180 patients within 30 months based on the annual number of RARPs performed in our hospital, accounting for a 15% withdrawal/refusal rate. No statistical sample size calculation was performed, because no previous evidence has been reported on the effect of frailty on postoperative pain. We enrolled 181 patients with localized PC who were treated at the Hirosaki University Hospital between January 2017 and June 2019. The inclusion criteria called for patients with localized PC who were treated with RARP. Procedures of RARP and standard pelvic lymph node dissection were performed using the basic technique described previously.^21^, ^22^ Extended pelvic lymph node dissection was not performed in this study. The exclusion criteria were as follows: (1) patients who could not be evaluated for frailty and postoperative pain and (2) those treated with open radical prostatectomy. All RARPs were mainly performed five expert surgeons who had enough experience of all urological surgeries and patients were equally distributed among surgeons.

### 2.3 Patient variables

The patient variables evaluated at diagnosis were age, sex, Eastern Cooperative Oncology Group performance status (ECOG PS), serum prostate‐specific antigen (PSA), hypertension (HTN), cardiovascular disease (CVD), diabetes mellitus (DM), chronic respiratory disease (CRD), instrumental activities of daily living (IADL), the American Society of Anesthesiologists (ASA) physical status classification score, Gleason score, clinical stage, and D’Amico risk classification. Postoperative complications were evaluated using Clavien–Dindo classification.

### 2.4 Assessment of frailty

We assessed frailty using the G8 screening tool and sFI (Figure S1). G8, which includes eight items in multiple geriatric assessment domains, was administered at the initial outpatient clinic visit. The G8 score ranges from 0 to 17, with a frailty cutoff of ≤14.^19^ We evaluated sFI including comorbidities (HTN, DM, CVD, CRD) and IADL. Non‐frail patients in sFI displayed none or one characteristic (score 0–1), whereas frail patients showed two or more characteristics (score 2–5). IADL was assessed using the Tokyo Metropolitan Institute of Gerontology Index of Competence (TMIG) index,^23^ with TMIG scores ranging from 1 to 13. Functional capacity impairment (IADL‐low) was assumed when a patient scored <11 (an IADL of < 80%).^4^

### 2.5 Evaluation of pain and use of analgesics

We used a numerical rating scale (NRS) for the quantitative evaluation of pain. NRS was measured on preoperative day 0, postoperative days 1, 2, 3, and at discharge as a self‐reported survey.^24^ The baseline NRS score was reported was prior to admission of any additional pain relief. Moderate to severe pain was defined as NRS ≥ 5.^25^, ^26^ We developed our original analgesic score for the postoperative use of pain relievers. We used acetaminophen (1000 mg, intravenously), loxoprofen sodium hydrate (60 mg, orally), and diclofenac (50 mg, suppository) as a standard initial dose. The number of analgesics was scored according to the amount and type of agent (standard initial dose of antianalgesic agent = score 1) used during postoperative periods. When a patient used acetaminophen 1000 mg twice, half dose of diclofenac 50 mg once, and loxoprofen sodium hydrate 60 mg once, the analgesics score was 3.5 (= 2 + 0.5 + 1) points. This scoring system was developed according to our previous study that evaluated the use of antihypertensive medications.^27^

### 2.6 Anesthetic procedure and intraoperative period

The anesthetic management and postoperative analgesia of patients was consistent and was not modified during this study. RARP was performed using general anesthesia, which consisted of remifentanil, ketamine, and propofol. At the end of the surgery, intravenous morphine (5‐10 mg) or fentanyl (2‐4 μg/kg) was administered as boluses for an opioid rotation. Thereafter, intravenous patient‐controlled analgesia using morphine (20‐30 mg/day) or fentanyl (400‐500 μg/day) with ketamine (20 mg/day) was administered to manage postoperative pain within 2 days. All patients used the same composition of patient‐controlled analgesia with a total volume of 50 ml for 48 hours.

### 2.7 Outcomes

The primary objectives of this study were to assess the effect of moderate to severe pain (NRS ≥ 5) on frailty, postoperative complications, and the use of analgesics after RARP. Secondary objectives were evaluating the effect of frailty on postoperative complications and the use of analgesics. The exploratory objectives included (1) the association between frailty and time‐dependent change of NRS, (2) the association between maximum NRS (maximum pain) and total NRS (total pain), (3) the optimal cutoff value evaluation of G8 for NRS ≥ 5 using the receiver operating characteristic (ROC) curve and area under the curve (AUC), and (4) the association between the G8 and sFI.

### 2.8 Statistical analysis

Statistical analysis was performed with GraphPad Prism 7.00 (GraphPad Software, Inc., San Diego, CA, USA), Bell Curve for Excel (Social Survey Research Information Co., Ltd., Tokyo, Japan), and R 3.3.3 (The R Foundation for Statistical Computing, Vienna, Austria). Categorical variables were compared using Fisher's exact test or the *χ* ^2^ test. Quantitative variables were expressed as means ± standard deviation. The significance of between‐group differences was determined using Student's *t* test for normally distributed data or the Mann–Whitney *U* test for nonnormally distributed data. A *P*‐value < .05 was considered statistically significant. Odds ratio (OR) with 95% confidence intervals (95% CI) were calculated using the multivariate logistic regression model for NRS ≥ 5 after controlling for potential confounders including age, frailty (G8 ≤ 14), analgesic score, and any complications.

## 3 RESULTS

### 3.1 Patient selection and characteristics

Of 181 patients, we excluded six patients who were treated with open radical prostatectomy and 21 patients who did not provide any response to the NRS questionnaire. This left us with a total of 154 patients for the analysis. The median age, PSA, G8, length of hospital‐stay, ASA score, and analgesic score were 69 years, 8.74 ng/mL, 14 days, 2, and 2, respectively. The number of patients who experienced grade 1, 2, and 3 complications were 19 (12%), 4 (2.6%), and 1 (0.7%), respectively. Of the 154 study participants, 37 (24%) and 61 (40%) patients were classified to have NRS ≥ 5 and G8 ≤ 14, respectively. The median NRS on days 0, 1, 2, 3, and at discharge were 0, 3, 2, 1, and 0, respectively. Meanwhile, the median value of total pain (sum of NRS) was 6, and the number of patients with moderate to severe pain (NRS ≥ 5) was 37 (24%). The maximum value of NRS was observed on postoperative day 1 in 153 (99.3%) patients. There was no significant difference in background between patients with NRS < 5 and those with NRS ≥ 5 (Table 1). The number of patients who underwent standard lymph node dissection was not significantly different between the NRS < 5 and NRS ≥ 5 (*P* = .487). Also, the complication rate was not significantly different between the patients with and without PLND (11% vs 20%, *P* = 0.075). The median total analgesic score in patients with NRS < 5 and those with NRS ≥ 5 was 2 (IQR: 1–4, range: 0–11) and 1 (IQR: 0–4, range 0–11) (*P* = 0.451).

**Table 1:** Background of participants

	NRS < 5	NRS ≥ 5	*P* value
Number of patients	117	37	
Age, years, median (IQR)	69 (65–71)	69 (64–72)	.856
ECOG PS, median (IQR)	0 (0–0)	0 (0–0)	1.000
Cardiovascular disease (CCVD), n	11 (9.4%)	4 (11%)	.758
Diabetes mellitus (DM), n	16 (14%)	5 (14%)	1.000
Hypertension (HTN), n	62 (53%)	21 (57%)	.073
Chronic respiratory disease (CRD), n	1 (0.9%)	1 (2.7%)	.424
Instrumental activities of daily living (IADL)‐low (<80%)	11 (9.4%)	2 (5.4%)	.735
ASA score, median (IQR)	2 (2–2)	2 (2–2)	.679
Gleason score, median (IQR)	7 (7–9)	7 (7–8)	.066
Initial PSA, ng/mL, median (IQR)	9 (5–12)	9 (5–12)	.145
D`Amico High‐risk, n	68 (58%)	18 (49%)	.346
Operation time (min)	164 (143–195)	178 (151–192)	.505
Blood loss (g)	25 (10–50)	25 (10–50)	.989
Standard pelvic lymph node dissection, n	50 (43%)	17 (46%)	.487
Total analgesic score, median (IQR)	2 (1–4)	1 (0–4)	.451
Hospital stay, days, median (IQR)	15 (14–15)	14 (14–15)	.395

### 3.2 Primary outcomes

**Figure 1:**
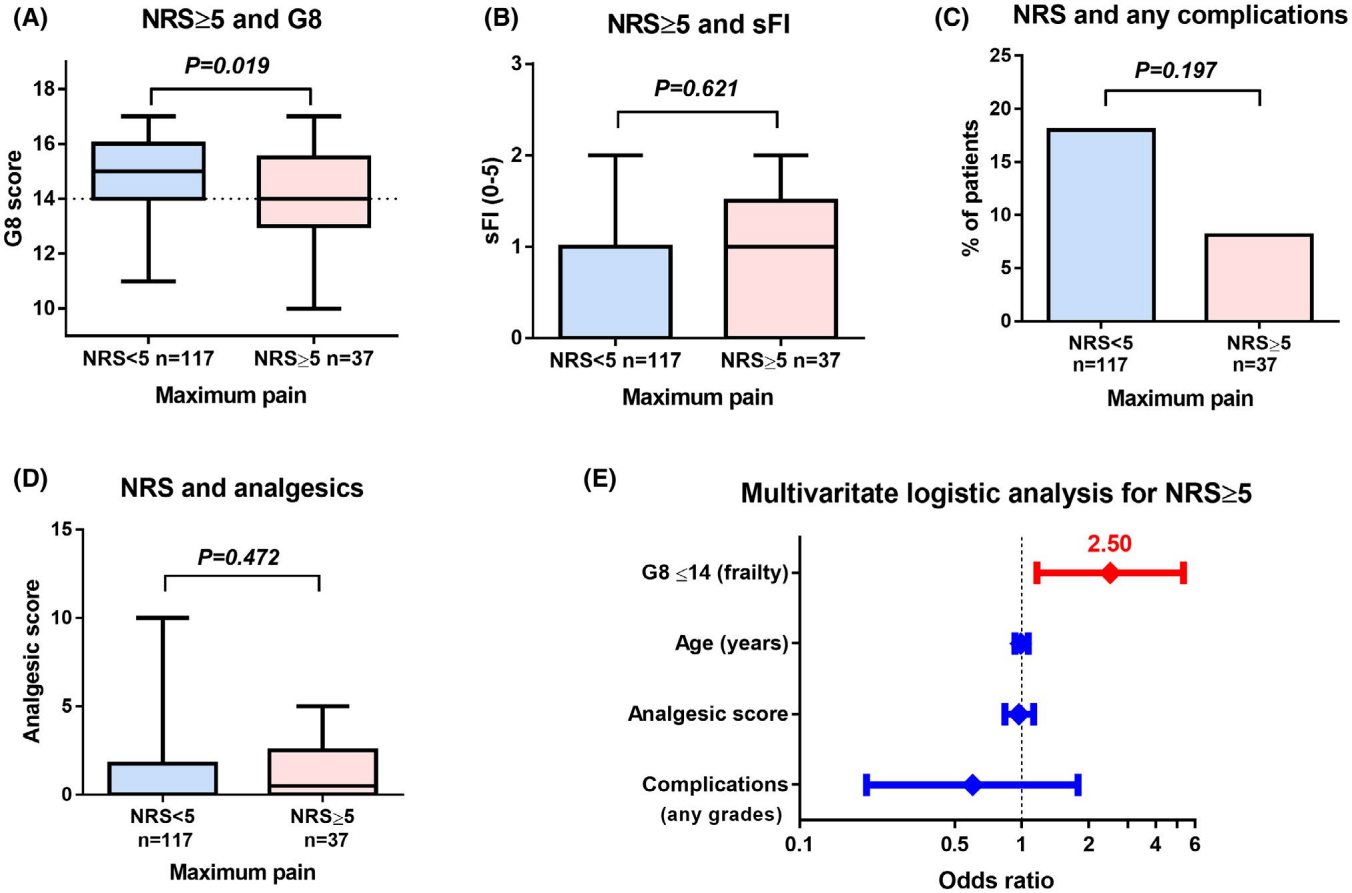
Primary outcomes measure. The effect of the numerical rating scale (NRS) on geriatric 8 (G8) (A), simplified frailty index (sFI) (B), postoperative complications (any grades) (C), and the use of analgesics (D) were investigated. Multivariate logistic regression analysis was performed to investigate the association of multiple variables on NRS ≥ 5 (E)

Patients with NRS ≥ 5 were significantly associated with G8 ≤ 14 than those with NRS < 5 (Figure 1A), whereas sFI (Figure 1B), postoperative complications (Figure 1C), and analgesics (Figure 1D) were not. The number of patients and the grade of postoperative complications are shown in Table 2. Multivariate logistic regression analysis showed that a G8 score of ≤ 14 was significantly associated with NRS ≥ 5 (Figure 1E and Table 3; *P* = .018, OR 2.50, 95% CI 1.17‐5.33).

**Table 2:** Postoperative complications

	NRS < 5	NRS ≥ 5	*P* value
Any complications	21 (18%)	3 (8.1%)	.197
Grade 1	18	1	
Grade 2	2	2	
Grade 3	1	0	

**Table 3:** Multivariate logistic regression analysis for NRS ≥ 5

Analysis for NRS ≥ 5		*P* value	OR	95%CI
Age	Continuous	.848	0.99	0.93–1.07
Frailty	G8 ≤ 14	.018	2.50	1.17–5.33
Any complications	Positive	.363	0.60	0.20–1.79
Analgesic score	Continuous	.735	0.97	0.84–1.13

### 3.3 Secondary outcomes

**Figure 2:**
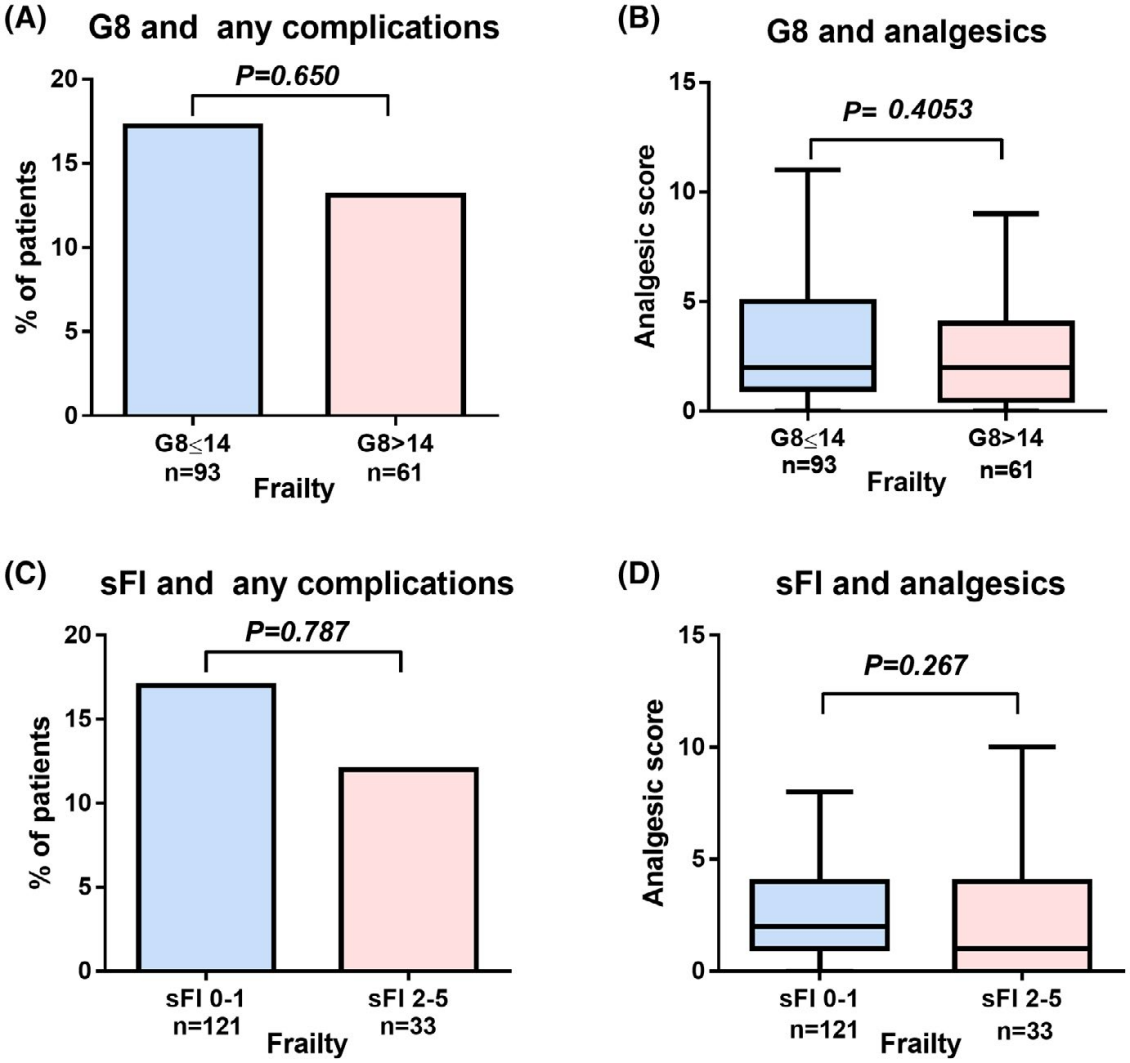
Secondary outcomes measure. The effect of geriatric 8 (G8) on postoperative complications (any grades) (A) and use of analgesics (B) were investigated. The effect of sFI 2–5 on postoperative complications (any grades) (C) and use of analgesics (D) were investigated

The G8 score was not significantly associated with postoperative complications (Figure 2A) and the use of analgesics (Figure 2B). The sFI 2‐5 was not significantly associated with postoperative complications (Figure 2C) and the use of analgesics (Figure 2D).

### 3.4 Exploratory outcome

**Figure 3:**
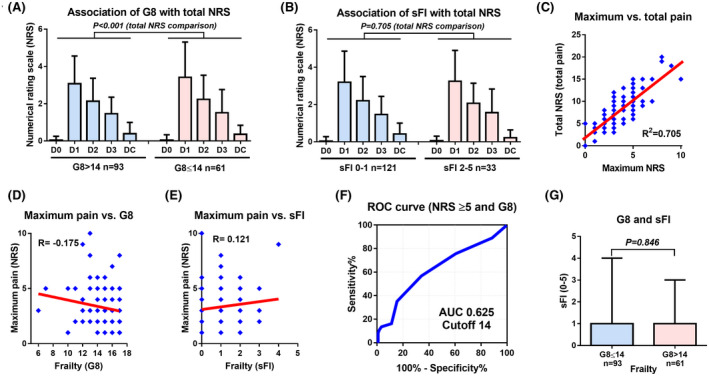
Exploratory outcome. The association between frailty and time‐dependent change of numerical rating scale (NRS) was investigated in geriatric 8 (G8) (A) and simplified frailty index (sFI) (B). The association between maximum NRS (maximum pain) and total NRS (total pain) was also evaluated (C). The linear association between the total NRS and maximum NRS (D), between maximum NRS and G8 (E), between maximum NRS and sFI (F) were evaluated. The optimal cutoff value of G8 for NRS ≥ 5 was evaluated using receiver operating characteristic (ROC) curve and the area under the curve (AUC) (E)

The time‐dependent change in NRS scores between G8 > 14 and ≤ 14 and between sFI 0–1 and 2–5 was shown in Figure 3A,B, respectively. Regarding frailty, patients with G8 ≤ 14 had significantly greater total pain (total NRS) than those with G8 > 14 (Figure 3A); meanwhile, the frailty of sFI was not significantly associated with total NRS (Figure 3B). Maximum NRS was significantly correlated with sum of NRS (*R* ^2^ = .705, *P* < .001) (Figure 3C). The linear association between the G8 and maximum NRS (Figure 3D) and between the sFI and maximum NRS (Figure 3E) was very weak (correlation coefficient: *R*, −.2 to.2). The optimal cutoff value of G8 for the NRS of ≥5 was defined as 14 with an AUC of 0.625 (Figure 3F). A G8 score of ≤14 was not significantly associated with sFI (Figure 3G). The linear association between the G8 and sFI was a very weak association (Figure S1C; correlation coefficient: *R*, −.2 to .2).

## 4 DISCUSSION

In this prospective observational study, we found that G8 ≤ 14 was an independent risk factor for postoperative moderate to severe pain after RARP. As our results suggested that use of analgesics and postoperative complications were not significantly different, these factors were potentially not associated with postoperative moderate to severe pain. Moreover, our results support the cutoff of G8 ≤ 14 for postoperative pain in patients with localized PC. To our knowledge, this is the first study to evaluate the effect of frailty on postoperative pain after RARP.

The key finding of our study was that patients with frailty might be sensitive to pain than those without frailty. We speculate that patients with painful experiences such as complications may require greater amounts of analgesics compared with patients without severe pain or complications. However, total analgesic score and complication rate were not significantly different between patients with NRS < 5 and those with NRS ≥ 5. These results may suggest that NRS reveals not only physical pain, but also psychological pain. However, our analgesic score was not validated and further study is necessary to address this issue. A cross‐sectional study suggested that a higher level of frailty is associated with a higher risk of adverse physical and psychological health situations.^28^ Another cross‐sectional study, which involved a total of 252 community‐dwelling elderly individuals (mean age, 79.2 ± 7.3 years), examined the relationship between frailty and pain.^29^ In that study, Coelho et al^29^ found that a greater pain impact score was associated with the presence of frailty (OR = 1.06; 95% CI, 1.03–1.10; *P* < .001). As frail patients do not have enough capacity to handle stress, they find it difficult to deal with painful postoperative experiences, which might be not as severe in patients without frailty. For this reason, intolerable pain may lead to difficulty in early ambulation and resumption of eating, thereby resulting in unfavorable postoperative outcomes for patients with frailty. Although longitudinal studies are required to help us understand the causal relationship between pain and frailty, pain might play a key role in frailty.

Although our study failed to show an association between frailty and incidence of postoperative complications after RARP, a previous study using the American College of Surgeons National Surgical Quality Improvement Program (ACS‐NSQIP) database (*n* = 23 104) suggested that there was a significant association between frailty (modified frailty index [mFI]) and postoperative Clavien–Dindo IV complications after RARP.^9^ The authors suggested that patients with frailty had increased rates of (any) complications, wound disruptions, bleeding transfusions, and 30‐day mortality. Patients with the highest mFI scores (≥3) had significantly higher rate of Clavien–Dindo IV complications (OR = 12.107; 95% CI, 2.800–52.351, *P* < .005) in comparison with patients without frailty. Therefore, RARP was not recommended for patients with frailty because of worsening outcomes. These findings suggested that frailty might be a useful tool in identifying the optimal patients for surgical interventions. Our previous study revealed that frailty was significantly higher in patients with muscle‐invasive bladder cancer who underwent nonsurgical therapy than in those who underwent radical cystectomy (frailty discriminant score: 3.27 vs 2.06, respectively; *P* < .001).^30^ This might be in line with clinical experience because we selected a noninvasive therapy for frail patients who were ineligible for surgical interventions. Therefore, frailty might be the key factor for the treatment selection to avoid severe postoperative complications and painful experiences. Further studies are necessary to address the effect of frailty on treatment selection in patients with localized PC.

Our results showed a significant correlation between maximum NRS and total NRS. This finding suggested that NRS evaluation on day 1 might be enough to estimate frailty‐related intolerability for pain because 99.3% of patients experienced maximum pain on postoperative day 1. G8 screening at admission and NRS assessment on postoperative day 1 might provide us with useful information for pain management. Because frail patients are sensitive to pain, those with G8 ≤ 14 can potentially experience severe pain postoperatively. To help mitigate this effect, we can modify the dose and timing of analgesics and opioids in such patients based on the NRS assessment on postoperative day 1. Although further studies are necessary, frailty assessment might be useful for postoperative pain management.

The reason for the no significant association of sFI with moderate to severe pain (NRS ≥ 5) after RARP needs to debate. As the sFI was the comorbidity‐based frailty, it was suggested that the significant association of comorbidity‐based frailty tools with severe (Clavien‐Dindo grade IV or V) postoperative complications.^8^, ^31^ As no grade IV or V complication was observed in this study, sFI might be not significantly associated with moderate to severe pain (NRS ≥ 5) after RARP.

We observed no significant association between frailty in G8 and sFI. Although both tools were developed for frailty measurement, consensus between different frailty assessment tools remains controversial to date. Our previous study comparing the Fried phenotype (FP) frailty, G8, and frailty discriminant score indicated fair agreement (*κ* = 0.381) among the three frailty tools.^4^ A systematic review, which investigated the sensitivity and specificity of the assessment tools for predicting frailty in elderly patients with cancer, also reported similar findings.^20^ Based on their analysis, van Walree et al. noted that the G8 screening tool has a high sensitivity for frailty (87%) but has poor specificity (61%). Conversely, the FP criteria have high specificity for frailty (91%) but have poor sensitivity (31%).^20^ They concluded that the use of a single frailty screening tool is not sufficient in identifying patients with frailty because of multidimensionality factors.^20^ As each tool evaluated different aspects of frailty, it might be not easy to find the “one size fits all” tool for frailty evaluation. Furthermore, optimal frailty tools may change depending on the type of disease. Therefore, a single frailty test might be not enough to evaluate the multidimensional phenomenon of frailty. Further studies are warranted to identify a suitable combination of frailty tools and diseases.

As several studies suggested G8 of ≤ 14 is the useful cutoff of frailty in many cancers,^20^, ^32^ we selected 14 as a cutoff in this study. However, the definition of the optimal cutoff of the G8 score remains unelucidated. Our recent study evaluating the association between the G8 and treatment by disease stages in a patient with PC suggests that the G8 ≤ 14 may be the useful cutoff of frailty between the surgical treatment (robot‐assisted radical prostatectomy) and non‐surgical therapies (radiotherapy and/or standard of care including androgen‐deprivation therapy) in patients with localized PC.^33^ Also, the poor prognosis was associated with G8 < 12 and <13 in the patients with metastatic castration‐sensitive and castration‐resistant prostate cancers, respectively.^33^ Therefore, the cutoff value of G8 may need to be modified depending on the disease status. Further studies are necessary to identify the optimal cutoff of G8 in the different disease stages.

The study limitations include the small sample size, selection bias, and unmeasurable confounding factors. We could not address the association of frailty with the type of pain because of data unavailability. The results may not be generalized to other countries because of racial and regional differences. For example, the median duration of hospital stay was not significantly different between patients with NRS < 5 and those with NRS ≥ 5 (15 vs 14 days, *P* = .395), because our national insurance system covers hospitalization costs. We could not select hospital stay as a primary endpoint because of the universal insurance system used in Japan. Also, the sample size was not enough to evaluate longitudinal changes over time as well as the aspect of confounding introduced by repeated measurements. Despite these limitations, the study demonstrates the clinical value of frailty on postoperative pain in patients with RARP. Our results suggested that frailty is a key factor for pain management in clinical practice. Further study is necessary to validate our findings.

In conclusion, frailty was significantly associated with moderate to severe pain after RARP and might be a potential predictor of postoperative pain. Frail patients require individual care to avoid painful postoperative experiences.

## CONFLICT OF INTERESTS

The authors have no conflicts of interest to declare.

## AUTHOR CONTRIBUTIONS

Conception and design: Shingo Hatakeyama, Chikara Ohyama; Acquisition of data: Masaki Momota, Osamu Soma, Naoki Fujita, Kyo Togashi, Tomoko Hamaya, Itsuto Hamano, Teppei Okamoto, Hayato Yamamoto, Tohru Yoneyama, Takahiro Yoneyama, Yasuhiro Hashimoto; Analysis and interpretation of data: Masaki Momota, Shingo Hatakeyama, Itsuto Hamano; Drafting of the manuscript: Masaki Momota, Shingo Hatakeyama; Critical revision of the manuscript: Chikara Ohyama; Statistical analysis: Shingo Hatakeyama, Tohru Yoneyama; Funding: Shingo Hatakeyama, Chikara Ohyama, Tohru Yoneyama; Administrative, technical, and material support: Tohru Yoneyama.

## References

[1] Townsend NT , Robinson TN . Surgical risk and comorbidity in older urologic patients. Clin Geriatr Med. 2015;31:591–601. 26476118 10.1016/j.cger.2015.06.009

[2] Isharwal S , Johanning JM , Dwyer JG , Schimid KK , LaGrange CA . Preoperative frailty predicts postoperative complications and mortality in urology patients. World J Urol. 2017;35:21–6. 27172940 10.1007/s00345-016-1845-z

[3] Suskind AM , Walter LC , Jin C , Boscardin J , Sen S , Cooperberg MR , Finlayson E . Impact of frailty on complications in patients undergoing common urological procedures: a study from the American College of Surgeons National Surgical Quality Improvement database. BJU Int. 2016;117:836–42. 26691588 10.1111/bju.13399 PMC4833543

[4] Soma O , Hatakeyama S , Okamoto T , Fujita N , Hamano I , Tanaka T , et al. Multicenter prospective study validating the efficacy of a quantitative assessment tool for frailty in patients with urological cancers. Med Oncol. 2019;13(36):88. 10.1007/s12032-019-1313-x 31520152

[5] Sato T , Hatakeyama S , Okamoto T , Yamamoto H , Hosogoe S , Tobisawa Y , et al. Slow gait speed and rapid renal function decline are risk factors for postoperative delirium after urological surgery. PLoS ONE. 2016;11:e0153961. 27145178 10.1371/journal.pone.0153961 PMC4856409

[6] Soma O , Hatakeyama S , Imai A , Matsumoto T , Hamano I , Fujita N , et al. Relationship between frailty and lower urinary tract symptoms among community‐dwelling adults. Low Urin Tract Symptoms. 2019;12:128–36. 31642610 10.1111/luts.12292

[7] Taylor BL , Xia L , Guzzo TJ , Scherr DS , Hu JC . Frailty and greater health care resource utilization following major urologic oncology surgery. Eur Urol Oncol. 2019;2:21–7. 30929842 10.1016/j.euo.2018.06.005

[8] Lascano D , Pak JS , Kates M , Finkelstein JB , Silva M , Hagen E , et al. Validation of a frailty index in patients undergoing curative surgery for urologic malignancy and comparison with other risk stratification tools. Urol Oncol. 2015;33(426):e1–12. 10.1016/j.urolonc.2015.06.002 PMC4584178 26163940

[9] Levy I , Finkelstein M , Bilal KH , Palese M . Modified frailty index associated with Clavien‐Dindo IV complications in robot‐assisted radical prostatectomies: a retrospective study. Urol Oncol. 2017;35:425–31. 28190748 10.1016/j.urolonc.2017.01.005

[10] Kakehi Y , Sugimoto M , Taoka R . Evidenced‐based clinical practice guideline for prostate cancer. Int J Urol. 2017;24:648–66. 28667698 10.1111/iju.13380

[11] Kimura T , Egawa S . Epidemiology of prostate cancer in Asian countries. Int J Urol. 2018;25:524–31. 29740894 10.1111/iju.13593

[12] Hatakeyama S , Yoneyama T , Tobisawa Y , Ohyama C . Recent progress and perspectives on prostate cancer biomarkers. Int J Clin Oncol. 2017;22:214–21. 27730440 10.1007/s10147-016-1049-y PMC5378754

[13] Ito K , Oki R , Sekine Y , Arai S , Miyazawa Y , Shibata Y , et al. Screening for prostate cancer: history, evidence, controversies and future perspectives toward individualized screening. Int J Urol. 2019;26:956–70. 31183923 10.1111/iju.14039

[14] Okamoto T , Hatakeyama S , Narita S , Arai Y , Habuchi T , Ohyama C . Validation and development of the CHAARTED criteria in patients with hormone‐naive metastatic prostate cancer: a multi‐institutional retrospective study in Japan. Int J Urol. 2020;27:90–1. 31617248 10.1111/iju.14136

[15] Culp MB , Soerjomataram I , Efstathiou JA , Bray F , Jemal A . Recent global patterns in prostate cancer incidence and mortality rates. Eur Urol. 2019;77:38–52. 31493960 10.1016/j.eururo.2019.08.005

[16] Molina‐Garrido MJ , Guillen‐Ponce C . Use of geriatric assessment and screening tools of frailty in elderly patients with prostate cancer. Rev Aging Male. 2017;20:102–9. 10.1080/13685538.2016.1277516 28084133

[17] Droz JP , Albrand G , Gillessen S , Hughes S , Mottet N , Oudard S , et al. Management of prostate cancer in elderly patients: recommendations of a task force of the international society of geriatric oncology. Eur Urol. 2017;72:521–31. 28089304 10.1016/j.eururo.2016.12.025

[18] Sathianathen NJ , Jarosek S , Lawrentschuk N , Bolton D , Konety BR . A simplified frailty index to predict outcomes after radical cystectomy. Eur Urol Focus. 2018;5:658–63. 29366857 10.1016/j.euf.2017.12.011

[19] Hamaker ME , Jonker JM , de Rooij SE , Vos AG , Smorenburg CH , van Munster BC . Frailty screening methods for predicting outcome of a comprehensive geriatric assessment in elderly patients with cancer: a systematic review. Lancet Oncol. 2012;13:e437–44. 23026829 10.1016/S1470-2045(12)70259-0

[20] van Walree IC , Scheepers E , van Huis‐Tanja L , Emmelot‐Vonk MH , Bellera C , Soubeyran P , et al. A systematic review on the association of the G8 with geriatric assessment, prognosis and course of treatment in older patients with cancer. J Geriatr Oncol. 2019;10:847–58. 31078444 10.1016/j.jgo.2019.04.016

[21] Matsumoto T , Hatakeyama S , Ookubo T , Mitsuzuka K , Narita S , Inoue T , et al. Cost‐effectiveness comparison between neoadjuvant chemohormonal therapy and extended pelvic lymph node dissection in high‐risk prostate cancer patients treated with radical prostatectomy: a multi‐institutional analysis. Med Oncol. 2017;31(34):190. 10.1007/s12032-017-1050-y 29090390

[22] Fujita N , Koie T , Hashimoto Y , Narita T , Tobisawa Y , Tanaka T , et al. Neoadjuvant chemohormonal therapy followed by robot‐assisted and minimum incision endoscopic radical prostatectomy in patients with high‐risk prostate cancer: comparison of perioperative and oncological outcomes at single institution. Int Urol Nephrol. 2018;50:1999–2005. 30229466 10.1007/s11255-018-1985-8

[23] Yoshida D , Ohara T , Hata J , Shibata M , Hirakawa Y , Honda T , et al. Dairy consumption and risk of functional disability in an elderly Japanese population: the Hisayama study. Am J Clin Nutr. 2019;1(109):1664–71. 10.1093/ajcn/nqz040 31075788

[24] Karcioglu O , Topacoglu H , Dikme O , Dikme O . A systematic review of the pain scales in adults: Which to use? Am J Emerg Med. 2018;36:707–14. 29321111 10.1016/j.ajem.2018.01.008

[25] Serlin RC , Mendoza TR , Nakamura Y , Edwards KR , Cleeland CS . When is cancer pain mild, moderate or severe? Grading pain severity by its interference with function. Pain. 1995;61:277–84. 7659438 10.1016/0304-3959(94)00178-H

[26] Shi Q , Mendoza TR , Dueck AC , Ma H , Zhang J , Qian Y , et al. Determination of mild, moderate, and severe pain interference in patients with cancer. Pain. 2017;158:1108–12. 28267060 10.1097/j.pain.0000000000000890

[27] Fujita N , Hatakeyama S , Yamamoto H , Tobisawa Y , Yoneyama T , Yoneyama T , et al. Implication of aortic calcification on persistent hypertension after laparoscopic adrenalectomy in patients with primary aldosteronism. Int J Urol. 2016;23:412–7. 26840556 10.1111/iju.13060

[28] Tse MM , Lai C , Lui JY , Kwong E , Yeung SY . Frailty, pain and psychological variables among older adults living in Hong Kong nursing homes: can we do better to address multimorbidities? J Psychiatr Ment Health Nurs. 2016;23:303–11. 27307261 10.1111/jpm.12303

[29] Coelho T , Paul C , Gobbens RJJ , Fernandes L . Multidimensional frailty and pain in community dwelling elderly. Pain Med. 2017;1(18):693–701. 10.1111/pme.12746 25800906

[30] Soma O , Hatakeyama S , Okamoto T , Fujita N , Matsumoto T , Tobisawa Y , et al. Clinical implication of a quantitative frailty assessment tool for prognosis in patients with urological cancers. Oncotarget. 2018;3(9):17396–405. 10.18632/oncotarget.24712 PMC5915123 29707115

[31] Chappidi MR , Kates M , Patel HD , Tosoian JJ , Kaye DR , Sopko NA , et al. Frailty as a marker of adverse outcomes in patients with bladder cancer undergoing radical cystectomy. Urol Oncol. 2016;34(256):e1–6. 10.1016/j.urolonc.2015.12.010 PMC4875870 26899289

[32] Takahashi M , Takahashi M , Komine K , Yamada H , Kasahara Y , Chikamatsu S , et al. The G8 screening tool enhances prognostic value to ECOG performance status in elderly cancer patients: a retrospective, single institutional study. PLoS ONE. 2017;12:e0179694. 28640844 10.1371/journal.pone.0179694 PMC5480957

[33] Momota M , Hatakeyama S , Soma O , Hamano O , Fujita N , Okamoto T , et al. Geriatric 8 screening of frailty in patients with prostate cancer. Int J Urol. 2020:27. 10.1111/iju.14256 32500621

